# Simulation of Patch Field Effect in Space-Borne Gravitational Wave Detection Missions

**DOI:** 10.3390/s25103107

**Published:** 2025-05-14

**Authors:** Mingchao She, Xiaodong Peng, Li-E Qiang

**Affiliations:** 1 National Space Science Center, Chinese Academy of Sciences, Beijing 100190, China; shemingchao22@mails.ucas.ac.cn; 2 School of Fundamental Physics and Mathematical Sciences, Hangzhou Institute for Advanced Study, UCAS, Hangzhou 310024, China; 3 University of Chinese Academy of Sciences, Beijing 100049, China

**Keywords:** space-borne gravitational wave detection, inertial sensors, acceleration noise, patch field effects, simulation algorithm, low spatiotemporal complexity

## Abstract

Space-borne gravitational wave detection missions demand ultra-precise inertial sensors with acceleration noise below $3\times 10^{-15}\;m/s^{2}/\sqrt{Hz}$. Patch field effects, arising from surface contaminants and nonuniform distribution of potential on the test mass (TM) and housing surfaces, pose critical challenges to sensor performance. Existing studies predominantly focus on nonuniform potential distributions while neglecting bulge effects (surface deformation caused by the adhesion of pollutants or oxides, production and processing defects, and other factors) and rely on commercial software with limited flexibility for customized simulations. This paper presents a novel boundary element partitioning and octree-based simulation algorithm to address these limitations, enabling efficient simulation of both electrostatic and geometric impacts of patch fields with low spatiotemporal complexity (O(n)). Leveraging this framework, we systematically investigate the influence of single patches on the TM electrostatic force ($\Delta F_{x}$) and stiffness ($\Delta K_{xx}$) through parametric studies. Key findings reveal that $\Delta F_{x}$ and $\Delta K_{xx}$ exhibit linear dependence on patch potential variation (Δu) and can be fitted by a quartic polynomial (which can be simplified in some cases, such as only a cubic term) about patch radius (rₘ). The proposed method’s capability to concurrently model geometric bulges and potential nonuniformity offers significant advantages over conventional approaches, providing critical insights for gravitational wave data analysis. These results establish a foundation for optimizing mitigation strategies against patch-induced noise in future space missions.

## 1. Introduction

The detection of gravitational waves has revolutionized astrophysics since the first direct observation of a binary black hole merger by LIGO in 2015 [1]. This breakthrough opened a new window for exploring the universe, particularly in the mid- and low-frequency bands, which are best observed from space using laser interferometry [2,3,4,5,6]. To explore these frequency bands, several space-based missions have been proposed, including LISA [7], Taiji [8], and Tianqin [9]. These missions aim to achieve unprecedented sensitivity, enabling the detection of gravitational waves from sources such as supermassive black hole mergers and compact binary systems. Inertial sensors are critical components of space gravitational wave detectors, featuring a sensitive structure composed of a test mass (TM) and a housing equipped with electrodes. These electrodes include injection electrodes and sensing/actuation electrodes for position measurement and feedback control [10]. Space gravitational wave detection demands extremely low acceleration noise levels (e.g., $3\times 10^{-15}\;m/s^{2}/\sqrt{Hz}$ or better) [11,12]. To achieve this, it is essential to analyze and mitigate all potential noise sources, including those arising from patch fields of the inertial sensor.

Patch fields can arise on the surface of the TM or the inner surface of the housing due to factors such as surface contaminants, nonuniform material segregation, and variations in crystal orientation [13,14,15,16,17,18]. Patches lead to nonuniform potential distributions and localized bulges (surface deformation caused by the adhesion of pollutants or oxides, production and processing defects, and other factors), which can significantly affect sensor performance by introducing unwanted forces and stiffness changes. Numerous studies have been conducted on the patch field effect of inertial sensors in space gravitational wave detection missions [13,19,20,21,22,23,24]. These studies can be separated into two approaches: theoretical derivation and experimental investigations. The experimental approaches are further divided into physical experiments and computer simulations. Existing studies mainly focus on the effect of nonuniform potential distribution caused by patches, ignoring the effect of bulges (surface deformation). Additionally, many simulation-based studies rely on commercial software, which limits the flexibility (such as using GPU, convenient simulation of patches, and so on) to design customized algorithms for unique requirements.

In this paper, we propose a novel numerical simulation algorithm based on boundary element partitioning and octree structure. This method enables the efficient simulation of patch field effects under various conditions with reduced computational complexity. Using this approach, we investigate the impact of a single patch on the force and stiffness of the TM. The remainder of this paper is organized as follows: Section 2 presents the mathematical model and simulation method for patch field analysis, including the subdivision of surfaces and the calculation of electric field forces. Section 3 introduces a low-complexity algorithm based on charge equivalence and octree structure to improve computational efficiency. Section 4 details the experimental setup, results, and discussion, focusing on the effects of patch size, location, and potential distribution. Finally, Section 5 concludes the paper with a summary of key findings and potential future research directions.

## 2. Mathematical Modeling of Patch Field Simulation

### 2.1. Surface Subdivision and Electric Field Modeling

**Figure 1:**
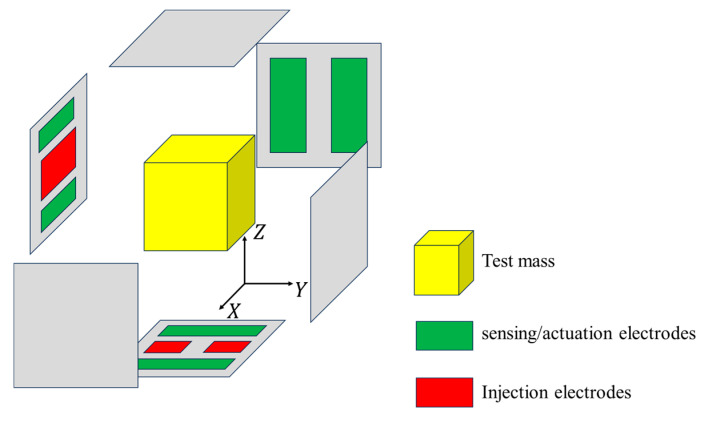
Schematic diagram of the sensitive structure.

**Figure 2:**
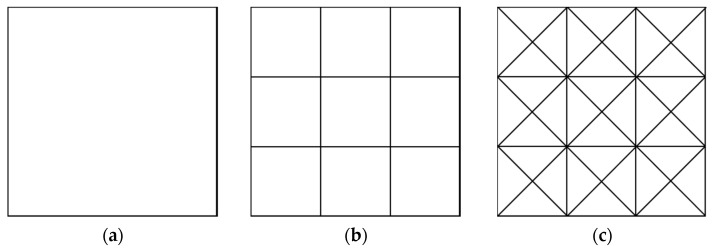
One example of subdividing a rectangular area. (**a**) The original rectangle; (**b**) some small rectangles divided by the original large cube; (**c**) triangular elements divided by those small rectangles.

**Figure 3:**
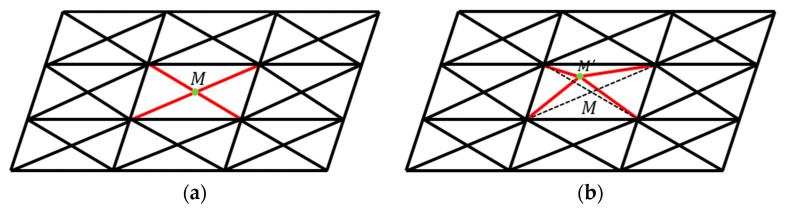
A method to create a bulge. (**a**) A rectangular area divided into several triangular elements; (**b**) one bulge will form after moving point *M* into $M^{\prime }$. Red lines: The triangular elements’ edges that are about to or have already changed. Dashed lines: The state of the changed edges before the change.

Figure 1 illustrates the schematic diagram of the sensitive structure. We define a rectangular coordinate system in space. With the housing as the reference frame, the geometric center of the housing is located at the origin, each face is perpendicular to a coordinate axis, and the x-axis direction coincides with the sensitive axis direction. When the TM is in equilibrium, its geometric center is also at the origin, and each face of the TM is also perpendicular to a coordinate axis. The surfaces of both the TM and housing are discretized into multiple rectangular regions. Each rectangular region is further subdivided into smaller rectangles, which are then divided into four triangular elements, as illustrated in Figure 2. This fine-grained subdivision enables precise modeling of surface geometry and facilitates the simulation of localized features, such as bulges caused by contaminants. By leveraging the geometric flexibility of triangles, we can simulate bulges by displacing specific nodes, as demonstrated in Figure 3.

#### 2.1.1. Potential and Charge Distribution

According to [25,26], the potential of the TM is

(1) $$u_{TM}=\frac{Q_{TM}+\sum_{k}C_{k}V_{k}}{\sum_{k}C_{k}},$$

where $Q_{TM}$ represents the charge accumulated on the TM due to cosmic rays, $C_{k}$ is the capacitance between the TM and a portion of the housing, and $V_{k}$ is the potential of the corresponding electrode. If the portion is not an electrode, $V_{k}$ is set to zero, as the area can be approximated as ground.

The potential at the centroid of each triangular element is assigned based on its location. For instance, if one triangular element whose centroid lies on an electrode with a potential of 3 mV, the potential of the triangular element is set as 3 mV. The potential at the centroid of triangular element *i* due to triangular element *j* is given by:

(2) $$u_{ij}=q_{j}\iint_{S_{j}}\frac{1}{4\pi r_{i}\left(Q\right)}dS_{j}\left(Q\right),$$

where $q_{j}=\frac{\lambda_{j}}{\epsilon_{0}}$, $\lambda_{j}$ is the charge surface density, and $\epsilon_{0}$ is the vacuum dielectric constant. $S_{j}$ is the area of the triangular element *j*, and $r_{i}\left(Q\right)$ stands for the distance between point *Q* and the centroid of the triangular element *i*. The potential at the centroid of triangular element *i* is the sum of contributions from all triangular elements:

(3) $$u_{i}=\sum_{j=1}^{n}u_{ij}=\sum_{j=1}^{n}q_{j}\iint_{S_{j}}\frac{1}{4\pi r_{i}\left(Q\right)}dS_{j}\left(Q\right),$$

where *n* stands for the number of triangular elements. For each triangular element, its $q_{j}$ is unknown, while the potential at its centroid can be obtained directly. We can construct a system of linear equations e.g., Equation (4) to solve the charge distribution:

(4) $$\left[\begin{array}{cccccc} g_{11} & g_{12} & \ldots & g_{1j} & \ldots & g_{1n}\\ g_{21} & g_{22} & \ldots & g_{2j} & \ldots & g_{2n}\\ \vdots & \vdots & \ddots & \vdots & \ddots & \vdots \\ g_{i1} & g_{i2} & \ldots & g_{ij} & \ldots & g_{in}\\ \vdots & \vdots & \ddots & \vdots & \ddots & \vdots \\ g_{n1} & g_{n2} & \ldots & g_{nj} & \ldots & g_{nn} \end{array}\right]\left[\begin{array}{c} q_{1}\\ q_{2}\\ \vdots \\ q_{j}\\ \vdots \\ q_{n} \end{array}\right]=\left[\begin{array}{c} u_{1}\\ u_{2}\\ \vdots \\ u_{i}\\ \vdots \\ u_{n} \end{array}\right]$$

The matrix element $g_{ij}$ is defined as:

(5) $$g_{ij}=\iint_{S_{j}}\frac{1}{4\pi r_{i}\left(Q\right)}dS_{j}\left(Q\right),$$

where $S_{j}$ is the area of triangular element *j*, and $r_{i}\left(Q\right)$ is the distance between point Q on triangular element *j* and the centroid of triangular element *i*. Since the value of *n* can be very large, we cannot solve Equation (4) directly. Instead, we employ the Generalized Minimal Residual (GMRES) method, an iterative solver well-suited for large linear systems.

#### 2.1.2. Calculation of Electric Field Force and Stiffness

In a moment, once the charge distribution is worked out, the electrostatic force and its stiffness acting on the test mass can be calculated. Specifically, we focus on $F_{x}$, which stands for the electrostatic force acting on the TM along the *X*-axis direction and the electrostatic stiffness $K_{xx}$, which is defined as the rate of change of $F_{x}$ with respect to the displacement of the TM along the *X*-axis. These quantities can be expressed as:

(6) $$F_{x}=\sum_{i=1}^{n_{TM}}\lambda_{i}\iint_{S_{i}}\sum_{j=n_{TM}+1}^{n}q_{j}\iint_{S_{j}}\frac{P_{x}-Q_{x}}{4\pi r^{3}\left(P,Q\right)}dS_{j}\left(Q\right)dS_{i}\left(P\right),$$

(7) $$K_{xx}=\sum_{i=1}^{n_{TM}}\lambda_{i}\iint_{S_{i}}\sum_{j=n_{TM}+1}^{n}q_{j}\iint_{S_{j}}\frac{r^{3}\left(P,Q\right)-3{\left(P_{x}-Q_{x}\right)}^{2}}{4\pi r^{5}\left(P,Q\right)}dS_{j}\left(Q\right)dS_{i}\left(P\right),$$

where $r\left(P,Q\right)$ stands for the distance between points *P* and *Q*, and $P_{x}$ and $Q_{x}$ represent the *x* coordinates of points *P* and *Q*, respectively. The number of triangular elements on the TM is $n_{TM}$, and of all the triangular elements (on all faces of the TM and the housing, a total of 12), the first $n_{TM}$ are on the TM.

#### 2.1.3. Algorithm Complexity

Storing the information of triangular elements requires a space complexity of $O\left(n\right)$. Due to the large value of *n*, the matrix in Equation (4) cannot be stored entirely. For example, if $n=10^{6}$ (the calculation accuracy is very low under such subdivision scale) and matrix elements are stored using 64-bit variables, the storage required to store the entire matrix will be about 7450.6 GB. Because of the extremely large storage requirement, matrix elements are computed on-the-fly when needed. The space complexity for calculating potential, force, and stiffness is $O\left(1\right)$.

When solving the equations iteratively, the time complexity required for one iteration of the solver is $O\left(n^{2}\right)$, and the same complexity applies to the calculation of electrostatic force and stiffness. The high time complexity limits the algorithm’s applicability, as it can only handle problems with relatively small values of *n*. Therefore, a low-complexity calculation method is urgently needed.

### 2.2. Simulation of a Patch

We first define the outer normal direction. At any point on a surface, the outer normal direction is perpendicular to the surface and points toward the gap between the housing and the TM. For the outer surface of the TM, the outer normal direction points to the outside of the TM, while for the inner surface of the housing, the outer normal direction points inward.

To simulate the single bulge, we introduce two parameters: $r_{m}$ (the base radius) and $d_{m}$ (the maximum displacement). A center point is selected, and all the nodes with a distance $\;r{<r}_{m}$ from the center are displaced along the outer normal direction by a distance *d*. The relationship between *r* and *d* is given by:

(8) $$r_{m}d+d_{m}r=r_{m}d_{m}.$$

**Figure 4:**
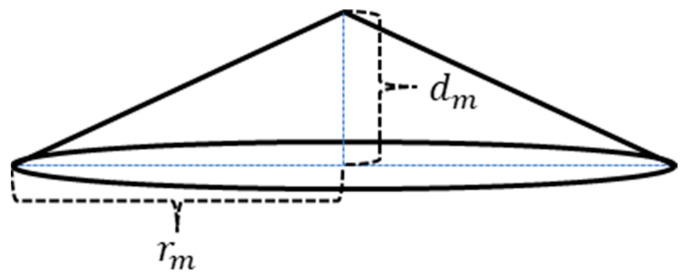
A patch bulge with a base radius of $r_{m}$ and a height of $d_{m}$.

Equation (8) and Figure 4 illustrate the typical shape of the bulge, which resembles a cone with a base radius of $r_{m}$ and a height of $d_{m}$. Specifically, if $d_{m}=0$, the patch affects only the potential distribution without altering the surface geometry.

All the triangular elements that have at least one vertex belonging to the nodes of the patch are considered part of the patch. Each triangular element of the patch involves the same change in the amount of the potential of the centroid of the triangular element. The influence of the parameters, such as $r_{m}$ and $d_{m}$, on the force and stiffness is investigated in detail in Section 4.

## 3. Low-Complexity Calculation Method

As mentioned in Section 2.1.3, we need to find an efficient algorithm to reduce computational complexity for large-scale simulations. Ying, L. has proposed a fast multipole method for particle simulations [27]. Many other improved algorithms [28,29,30] based on this method have also been developed. These algorithms inspired us. Aiming at the problems studied in this paper, we have made modifications and optimizations on the basis of these algorithms. This section will elaborate on the modified method and its application in our research.

### 3.1. The Theoretical Basis of Charge Equivalence

**Figure 5:**
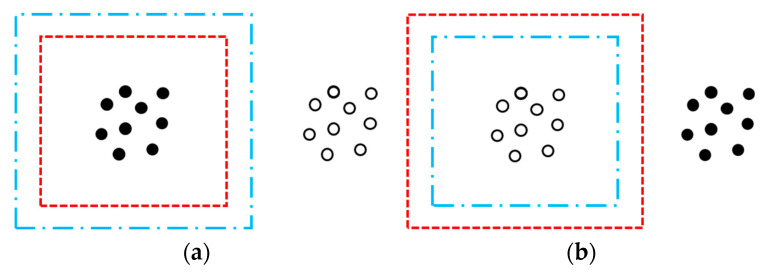
Schematic drawing of charges equivalent. Filled points: Charges to be equated. Hollow points: Points to be analyzed. Red dotted lines: The area where the charges to equate are located. Blue dashed lines: The boundary of the area described by Laplace’s equation. (**a**) Substitute the charges on the red boundary for the charges inside it to analyze the points outside the blue boundary; (**b**) substitute the charges on the red boundary for the charges outside it to analyze the points inside the blue boundary.

For the Laplace equation $\nabla^{2}u=0$, which governs potential distributions in singularity-free regions, the first kind of boundary conditions can be used as a complete definite solution condition [31]. For an area without any singularity inside (or outside) it, if two groups of charges outside (or inside) the region produce the same potential at any point on the border of the area, the effects of the two groups on the inside (or outside) of the area are equivalent. See Figure 5.

To discretize the problem, we employ *m* sampling points distributed along the region’s boundary. Let the charge distribution outside (or inside) the region be represented by a charge vector $S\in R^{n}$. The resultant electric potential at these sampling points can be formulated as $TS\in R^{m}$, where $T\in R^{m\times n}$ denotes the potential coefficient matrix mapping charges to potentials. To reconstruct the electric field inside (or outside) the region, we introduce an equivalent charge vector $Q\in R^{k}$ comprising *k* charges and a corresponding potential mapping matrix $E\in R^{m\times k}$, where $EQ\in R^{m}$ provides the potential vector at the sampling points. Then we can obtain

(9) EQ=TS.

If *E* is invertible, then

(10) $$Q=E^{-1}TS,$$

otherwise

(11) $$Q=E^{+}TS,$$

where $E^{+}$ is the generalized inverse of *E*. The process of multiplying *T* and ***S*** is called the first stage of the equivalency, and the process of multiplying $E^{-1}$ (or $E^{+}$) and *T**S*** is called the second stage of the equivalency.

### 3.2. Building an Octree

**Figure 6:**
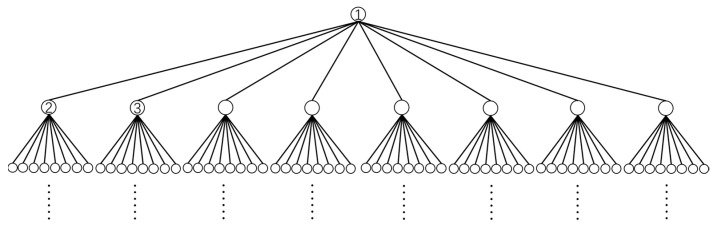
An octree. Each node has at most one parent node and eight children nodes. Node ① is the parent node of nodes ② and ③. Both nodes ② and ③ are the children nodes of ①. Those nodes that have no child node are leaf nodes. Node ①, which has no parent node, is the root node.

For a three-dimensional region $\left[x_{min},x_{max}\right]\times \left[y_{min},y_{max}\right]\times \left[z_{min},z_{max}\right]$, we can divide it into eight small regions listed in Table 1, where $x_{0}=\frac{x_{min}+x_{max}}{2}$, $y_{0}=\frac{y_{min}+y_{max}}{2}$, and $z_{0}=\frac{z_{min}+z_{max}}{2}$. After executing this process recursively, we can obtain an octree (see Figure 6). The recursive exit is when the volume of the current region is less than the threshold.

A pair of nodes are neighbors if and only if they are in the same layer of the octree and have common vertices, including the case where two nodes are the same node.

A pair of nodes are interactive nodes of the same layer if and only if their parent nodes are neighbors but they are not neighbors themselves.

**Figure 7:**
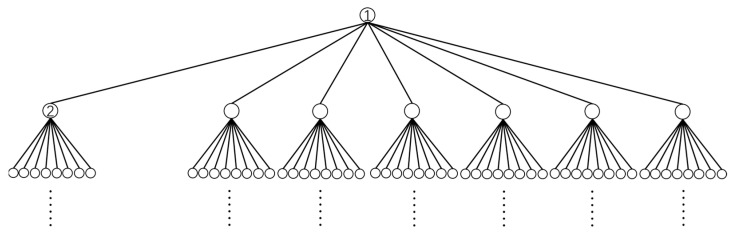
An octree after pruning. Node ③ in the octree in Figure 6 does not contain any triangular element, so it has not been divided and has been removed.

We chose a cube with edges of length *L* whose centroid is at (0, 0, 0), which can surround the whole sensitive structure. We constructed an octree recursively with this cube as the root node. To ensure each octree node contains at least one triangular element, the octree should be pruned. If one region is inside the TM, or outside the housing, or in the gap between the TM and the housing, we will not continue to divide it, and the node will not be considered, such as in Figure 7.

For each node of the octree, we built two auxiliary surfaces, scaling up with the centroid of the node as the center. According to [27], the proportional coefficients can be determined as $\frac{4}{3}$ and $\frac{7}{3}$, respectively. We refer to the inner (outer) one of the two surfaces as the inner (outer) auxiliary surface.

When analyzing the influence on the area outside the outer auxiliary surface of a node made by the charges inside the node, we should reconstruct the electric field outside the outer auxiliary surface by placing charges (equivalent point charges, defined in Section 3.1) on the inner auxiliary surface. The sampling points (defined in Section 3.1) are on the outer auxiliary surface.

When analyzing the influence on the area inside the inner auxiliary surface of a node made by part of the charges outside the outer auxiliary surface, we should reconstruct the electric field inside the inner auxiliary surface by placing charges on the outer auxiliary surface. The sampling points are on the inner auxiliary surface.

**Figure 8:**
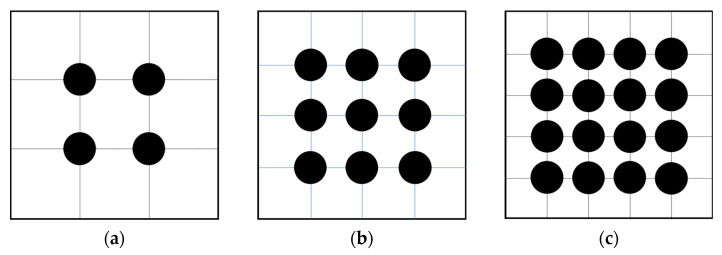
The distribution of charges or sampling points under different values of *p*. The grid lines are auxiliary lines and nonexistent in face. (**a**) p=2; (**b**) p=3; (**c**) p=4.

The placement of equivalent point charges and sampling points on the two kinds of auxiliary surfaces should be regular to facilitate the design of the algorithm. Each auxiliary surface is made up of six faces. We use the parameter *p* to describe the number of sampling points or charges. For one of the six faces, the number of sampling points or charges is $p^{2}$. Each face is divided into $\left(p+1\right)\times \left(p+1\right)$ regions by an imaginary grid, and the sampling points or charges are distributed on the grid nodes. For example, Figure 8 shows the distribution on a single face when p=2,3,4.

### 3.3. The Operation Performed on the Octree

**Figure 9:**
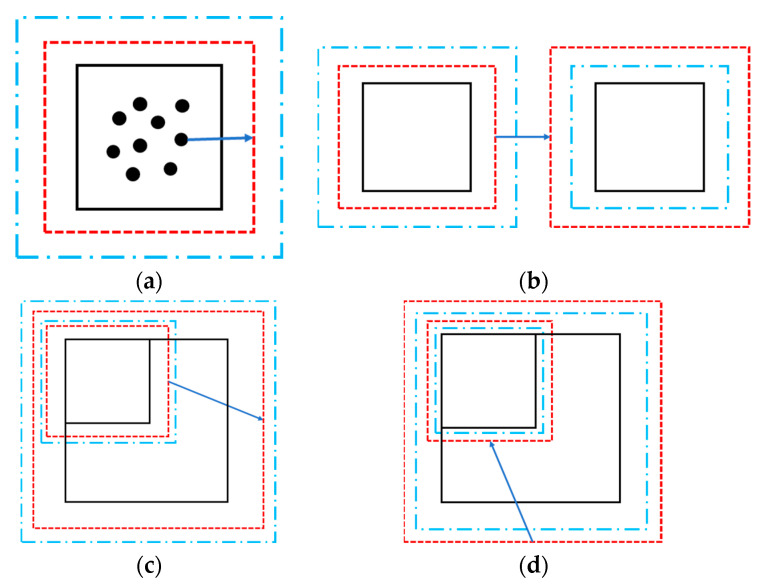
Schematic diagram of operations. Black points: Triangular elements. Black borders: The boundary of one node. Red dotted lines: The auxiliary used to lie charges. Blue dashed lines: The auxiliary used to lie sampling points. (**a**) Equivalenting source operation; (**b**) horizontal operation; (**c**) up operation; (**d**) down operation.

Here, we define four kinds of operations, including the equivalenting source operation, up operation, down operation, and horizontal operation. Figure 9 shows the schematic diagram of these operations. Among these operations, the equivalenting source operation and up operation are essentially the same as S2M and M2M in [27], while the down operation and horizontal operation are improved upon L2L and M2L in [27].

**Equivalenting source operation:** For a leaf, using the charges on its inner auxiliary surface to be equivalent to those triangular elements inside it and calling this process the equivalenting source operation for the leaf. The sampling points lie on the outer auxiliary surface of the leaf.

**Up operation:** For a node, excluding the root node, using the charges on its parent’s inner auxiliary surface to be equivalent to the charges on its inner auxiliary surface and calling this process the up operation for the node. The sampling points lie on the outer auxiliary surface of the node’s parent.

**Down operation:** For a node, excluding the root node, using the charges on its outer auxiliary surface to be equivalent to the charges on its parent’s outer auxiliary surface and calling this process the down operation for the node. The sampling points lie on the inner auxiliary surface of the node.

**Horizontal operation:** For a node *A* and a node *B*, which is one of the interactive nodes of the same layer of *A*, using the charges on the outer auxiliary surface of *A* to be equivalent to the charges on the inner auxiliary surface of *B* and calling this process the horizontal operation from *B* to *A*.

### 3.4. Main Body of the Algorithm

#### 3.4.1. Calculating Equivalent Charges

The general flow of calculating equivalent charges is to iterate from bottom to top first, calculate the charges on the inner auxiliary surfaces, and then iterate from top to bottom, and calculate the charges on the outer auxiliary surfaces. See Algorithm 1 for details.
**Algorithm 1.** Calculating equivalent charges**Input**: Constructed octree, needed matrixes, triangular elements information**Output**: The equivalent charges on two auxiliary surfaces for each node1**parfor** each leaf, *i*2  Executing the first and second steps of equivalenting source operation for *i* in sequence3**end parfor**4**for** *i* = *tree_hight* : 25  **parfor** each node, *j*, in layer *i* of the octree6    Executing up operation for *j*7  **end parfor**
11**end for**12**for** *i* = 2 : *tree_height*13  **parfor** each node, *j*, in layer *i* of the octree14    **for** each interactive node of the same layer, *k*, of *j*15      Executing horizontal operation from *k* to *j*16    **end for**
17  **end parfor**
18  parfor each node, j, in layer i+1(if it exists) of the octree19    Executing down operation for *j*20  **end parfor**
24**end for**25**return** the equivalent charges on two auxiliary surfaces for each node

The reason why we split the equivalenting source operation (line 2) into two steps instead of executing the whole directly will be explained in Section 3.5.1.

Different from calculating potential distribution, when obtaining force and stiffness, we only need the electric field provided by those triangular elements on the housing. So, we should rebuild the octree after we set $q_{i}=0$ for each $i\leq n_{TM}$.

To accelerate the calculation, we can adopt multi-dimensional GPU parallel computing. The starting node and ending node of each operation, as well as the matrix operation corresponding to the operation, can all correspond to different dimensions of multi-dimensional threads.

#### 3.4.2. Updating Expressions

Due to the equivalence of charges on triangular elements, those relevant expressions in Section 2 are no longer applicable and need to be modified accordingly. $N_{i}$ stands for the set composed of subscripts of the triangular elements in all neighbor nodes of the leaf where the *i*-th triangular element is located, and $C_{i}$ stands for the list composed of the ratio of charge quantity to vacuum dielectric constant of each equivalent charge on the outer auxiliary surface of the leaf where the *i*-th triangular element is located. In addition, $L_{i}$ stands for the set composed of the positions of each equivalent charge on the outer auxiliary surface of the leaf where the *i*-th triangular element is located. The expression from Equation (3) that describes the potential at the centroid of the *i*-th triangular element should be modified to

(12) $$u_{i}=\sum_{j\in N_{i}}q_{j}\iint_{S_{j}}\frac{1}{4\pi r_{i}\left(Q\right)}dS_{j}\left(Q\right)+\sum_{j=1}^{6p_{equ}^{2}}C_{i}\left(j\right)\frac{1}{4\pi r_{i}\left(L_{i}\left(j\right)\right)},$$

where $p_{equ}$ is the value of parameter *p* for the equivalent charges of the four kinds of operations.

If $N_{i}^{*}$ is the set formed by the subscripts of the triangular element located on the housing in all the neighboring nodes of the leaf where the *i*-th triangular element is located, and $C_{i}^{*}$ is the list formed by the relative charge amounts of each equivalent charge on the outer auxiliary surface when the charge on the surface of the TM is set to 0, then, the expression (6), which describes the force on the TM in the X-axis direction, should be replaced by (13), and the expression (7), which describes the rate of change of the force on the TM along the x axis with respect to the displacement of the TM along the *x* axis, should be modified to (14).

(13) $$\begin{array}{c} F_{x}=\sum_{i=1}^{n_{TM}}\lambda_{i}\iint_{S_{i}}\sum_{j\in N_{i}^{*}}q_{j}\iint_{S_{j}}\frac{P_{x}-Q_{x}}{4\pi r^{3}\left(P,Q\right)}dS_{j}\left(Q\right)dS_{i}\left(P\right)+\\ \sum_{i=1}^{n_{TM}}\lambda_{i}\iint_{S_{i}}\sum_{j=1}^{6p_{equ}^{2}}C_{i}^{*}\left(j\right)\frac{P_{x}-L_{i}{\left(j\right)}_{x}}{4\pi r^{3}\left(P,L_{i}\left(j\right)\right)}dS_{i}\left(P\right), \end{array}$$

(14) $$\begin{array}{c} K_{xx}=\sum_{i=1}^{n_{TM}}\lambda_{i}\iint_{S_{i}}\sum_{j\in N_{i}^{*}}q_{j}\iint_{S_{j}}\frac{r^{3}\left(P,Q\right)-3{\left(P_{x}-Q_{x}\right)}^{2}}{4\pi r^{5}\left(P,Q\right)}dS_{j}\left(Q\right)dS_{i}\left(P\right)+\\ \sum_{i=1}^{n_{TM}}\lambda_{i}\iint_{S_{i}}\sum_{j=1}^{6p_{equ}^{2}}C_{i}^{*}\left(j\right)\frac{r^{3}\left(P,L_{i}\left(j\right)\right)-3{\left(P_{x}-{L_{i}\left(j\right)}_{x}\right)}^{2}}{4\pi r^{5}\left(P,L_{i}\left(j\right)\right)}dS_{i}\left(P\right). \end{array}$$

With the help of the octree, we can improve the efficiency of calculating $u_{i}$, $F_{x}$, and $K_{xx}$. Just like in Section 3.4.1, calculating $u_{i}$, $F_{x}$, and $K_{xx}$ can be regarded as performing matrix operations on each leaf node, and multi-dimensional GPU parallel computing can still be used for acceleration.

### 3.5. Algorithm Complexity

The volume of leaves is determined, so it can be approximated that the average amount of triangular elements,$\;n_{ele}$, inside each leaf is determined. The number of leaves is $n_{leaf}\approx \frac{n}{n_{ele}}$. According to the properties of the octree, the number of nodes is $n_{node}\approx \frac{8}{7}n_{leaf}$.

Let $p_{sam,leaf}$ stand for the value of parameter *p* for the sampling points of all the equivalenting source operations. The following analysis is based on the assumption that $p_{equ}$, $p_{sam,leaf}$, and $n_{ele}$ are constant.

#### 3.5.1. Space Complexity

Each operation has a corresponding matrix which can be translated into two matrixes corresponding to the first and the second step of the operation. Both the up operation and down operation have at most eight different relative positions of one node and its parent. The horizontal operation contains at most $7^{3}-3^{3}=316$ different relative positions of two nodes in the same layer of an octree [29]. The function $g\left(r\right)=\frac{1}{4\pi r}$ has the property that for each k,r≠0, $g(kr)=\frac{1}{k}g\left(r\right)$. Making use of this property, we can execute the up operation, down operation, and horizontal operation with $O\left(p_{equ}^{4}\right)=O\left(1\right)$ space complexity [29,30]. Because of the uncertain distribution of triangular elements in each leaf, if we want to store the matrixes of the equivalenting source operation, we need the space complexity of $O\left(n_{leaf}n_{ele}p_{equ}^{2}\right)=O\left(n\right)$. However, we can store the matrix of the second step of the equivalenting source operation with $O\left(p_{equ}^{2}p_{sam,leaf}^{2}\right)=O\left(1\right)$, and if we do not precompute the matrixes of the first step, we can also finish the equivalenting source operation with $O\left(1\right)$ space complexity. To optimize computational efficiency, we precompute the matrix of the second step, thereby circumventing the computationally intensive matrix inversion operation inherent in the solution procedure.

To store the information of triangular elements, the space complexity is $O\left(n\right)$. To store the information of the octree, the space complexity is $O\left(n_{leaf}+n_{node}\right)=O\left(n\right)$.

In summary, the total space complexity is $O\left(n\right)$.

#### 3.5.2. Time Complexity

For the convenience of analysis, the acceleration brought by the use of GPU parallel computing is not considered. First, we analyze the time complexity of calculating equivalent charges in one iteration. For each leaf, the corresponding equivalenting source operation should be executed exactly once in the whole procedure. So, the total time complexity of the equivalenting source operation is $O\left(n_{leaf}\left(n_{ele}p_{sam,leaf}^{2}+p_{equ}^{2}p_{sam,leaf}^{2}\;\right)\right)=O\left(n\right)$. For each node, the computational procedure requires a maximum of one up operation, one down operation, and $6^{3}-3^{3}=189$ times horizontal operation [30]. The total time complexity of the up operation, down operation, and horizontal operation is $O\left(n_{node}p_{equ}^{4}\right)=O\left(n\right)$, $O\left(n_{node}p_{equ}^{4}\right)=O\left(n\right),$ and $O\left(189n_{node}p_{equ}^{4}\right)=O\left(n\right)$, respectively. In summary, the total time complexity of calculating equivalent charges in one iteration is $O\left(n\right)$.

Second, we analyze the time complexity of calculating potential, force, and stiffness. Each node has at most 27 neighbors and $6p_{equ}^{2}$ charges on the outer auxiliary surface, so the total time complexity is $O\left(n_{leaf}\left(27n_{ele}^{2}+6p_{equ}^{2}n_{ele}\right)\right)=O\left(n\right)$.

## 4. Experiments, Results, and Discussion

### 4.1. Parameter Setting

**Figure 10:**
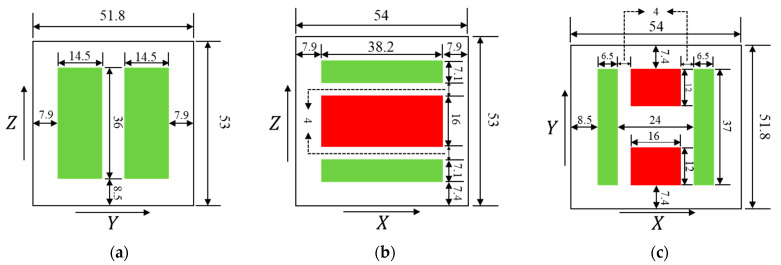
Electrode layout on the housing (Unit: mm). (**a**): Planar view at x=±27 mm. (**b**): Planar view at y=±25.9 mm. (**c**): Planar view at z=±26.5 mm.

For computational simplicity and analytical convenience, we model the TM as an ideal cube (with uniform side lengths of 46 mm), while the inner surface of the housing is approximated as an ideal cuboid surface. Based on the coordinate system defined in Section 2.1, Figure 10 shows the distribution of electrodes.

The red electrodes are injection electrodes, and the green electrodes are sensing and actuation electrodes. The other area of the surface of the housing can be approximated as grounding. According to Ref. [26], alternating current was applied to each electrode. We select a moment and assume that at that moment, the potential on all sensing electrodes is 1000 mV, and the potential on all injection electrodes is 3 mV. In addition, we assume $Q_{TM}=0$.

**Figure 11:**
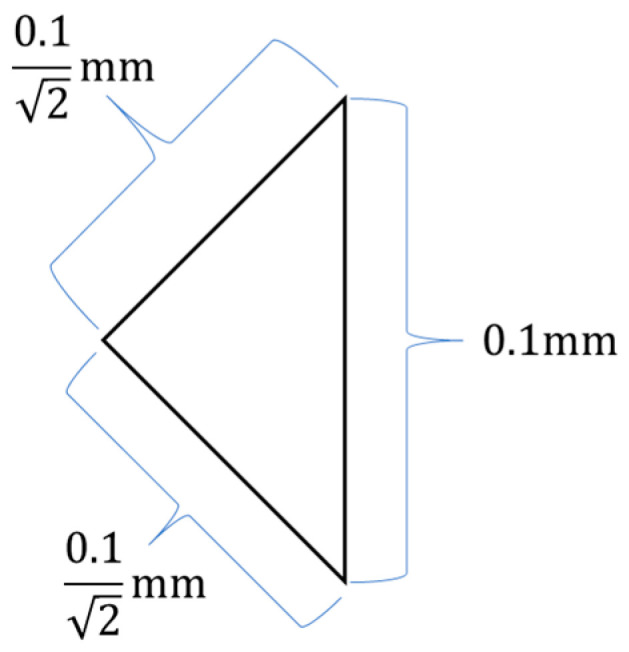
Fundamental subdivision unit—an isosceles right triangle with hypotenuse 0.1 mm.

To achieve surface subdivision, we first divide the domain into grids of squares with side length 0.1 mm; each square is subsequently divided into four triangular elements (as shown in Figure 11), forming the fundamental mesh unit. This process yields a total of 11,802,080 triangular elements.

### 4.2. Single Contaminant Patch Bulge Impact Analysis

In this section, we will analyze the influence of a single contamination patch bulge on the system, focusing on three key parameters: contaminant size, location, and potential variation. To streamline the analysis process, we adopt $d_{m}=\frac{1}{2}r_{m}$ in Equation (8), where $d_{m}$ represents the characteristic bulge height.

Before our experiments, we verified the correctness of our simulation algorithm. See Appendix A for detailed procedures.

To begin with, we calculated the electrostatic force $F_{x}^{0}\approx 3.72\times 10^{-19}\;N$ and the electrostatic stiffness $K_{xx}^{0}\approx -3.63\times 10^{-9}\;N/m$ under the circumstance that no patch exists. In theory, $F_{x}^{0}=0\;N$. The value of $3.72\times 10^{-19}\;N$ can be thought of as the error due to the subdivision, algorithm, and rounding error of floating-point numbers.

#### 4.2.1. Impact of Potential Δu

**Figure 12:**
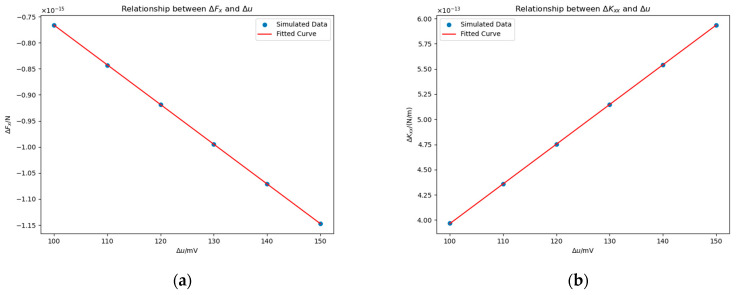
Impacts of Δu on $\Delta F_{x}$ (**a**) and $\Delta K_{xx}$ (**b**) in the presence of a contaminant bulge. Experimental parameters: The base radius of the contaminant bulge is $r_{m}=\frac{0.1}{1.415}\;mm$ and its position is at coordinates $\left(-23\;mm,\;0\;mm,\;0\;mm\right)$.

To characterize the electromechanical coupling effects under surface contamination, we conducted systematic variations of the electrostatic potential difference Δu from 100 mV to 150 mV with a step 10 mV. Throughout the experiments, the patch base radius $r_{m}$ was fixed at $\frac{0.1}{1.415}$ mm, and the center of the bulge’s base was positioned at the coordinate $\left(-23\;mm,\;0\;mm,\;0\;mm\right).$ As shown in Figure 12, a clear linear relationship is observed between $\Delta F_{x}$ and $\Delta K_{xx}$ and the potential difference Δ*u*. Notably, the electrostatic force change $\Delta F_{x}$ is defined as $\Delta F_{x}=F_{x}-F_{x}^{0}$, while the stiffness change $\Delta K_{xx}$ is characterized as $\Delta K_{xx}=K_{xx}-K_{xx}^{0}$.

We tried to fit the data using linear functions and obtained the following expressions

(15) $$\Delta F_{x}\approx \left(-7.62\Delta u-4.54\right)\times 10^{-18}\;N,$$

(16) $$\Delta K_{xx}\approx \left(3.94\Delta u+2.35\right)\times 10^{-15}\;N/m.$$

The actual meanings of their intercepts are the changes in the force or stiffness when the bulge has no influence on potential. Therefore, to reduce the error, we calculated the force and stiffness when the change in electric potential caused by the bulge was zero and assigned these values as the intercepts. During fitting, only the slope was adjusted.

#### 4.2.2. Impact of Base Radius $r_{m}$

**Figure 13:**
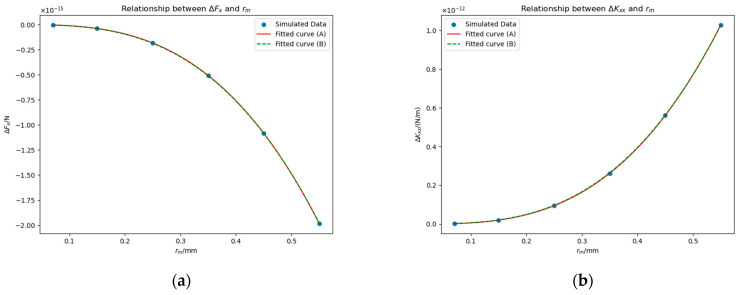
Impacts of $r_{m}$ on $\Delta F_{x}$ (**a**) and $\Delta K_{xx}$ (**b**) in the presence of a contaminant bulge. Simulation conditions: Δu=0 mV and the bulge is located at the coordinates $\left(-23\;mm,\;0\;mm,\;0\;mm\right)$.

To investigate the dependence of $\Delta F_{x}$ and $\Delta K_{xx}$ on the geometric scale of the bulge, we selected $r_{m}\in \left\{\frac{0.1}{1.415},0.15,0.25,0.35,0.45,0.55\right\}$ mm, set Δu=0, and chose point $\left(-23\;mm,\;0\;mm,\;0\;mm\right)$ as the center of the bottom of the bulge. Figure 13 illustrates the relationship between $\;r_{m}$ and the deviations $\Delta F_{x}$ and $\Delta K_{xx}$.

Theoretically, when $r_{m}=0\;mm$, both $\Delta F_{x}$ and $\Delta K_{xx}$ are expected to vanish, so we attempted to fit $\Delta F_{x}$ and $\Delta K_{xx}$ using cubic polynomials passing through the origin (see “Fitted curve (A)” in Figure 13). The resulting fitting expressions are:

(17) $$\Delta F_{x}\approx \left(-1.21r_{m}^{3}+8.31\times 10^{-3}r_{m}^{2}+6.61\times 10^{-4}r_{m}-1.03\times 10^{-4}\right)\times 10^{-14}\;N,$$

(18) $$\Delta K_{xx}\approx \left(6.33r_{m}^{3}-9.21\times 10^{-2}r_{m}^{2}+4.87\times 10^{-3}r_{m}+1.44\times 10^{-4}\right)\times 10^{-12}\;N/m.$$

In Equations (17) and (18), the coefficients of the cubic terms are several orders of magnitude larger than those of the remaining terms. Based on this observation, we inferred that $\Delta F_{x}\propto r_{m}^{3}$ and $\Delta K_{xx}\propto r_{m}^{3}$. To verify this conclusion, we re-fitted the data. The new fitting results are shown in “Fitted curve (B)” in Figure 13, and the formulas are:

(19) $$\Delta F_{x}\approx -1.19\times 10^{-14}r_{m}^{3}\;N,$$

(20) $$\Delta K_{xx}\approx 6.17\times 10^{-12}r_{m}^{3}\;N/m.$$

The fitting results confirm that $\Delta F_{x}$ and $\Delta K_{xx}$ exhibit a cubic dependence on $r_{m}$.

#### 4.2.3. Impact of Location

To further investigate the spatial dependence of contaminant path effects, we analyzed the positional sensitivity of patch effects by selecting four distinct bulge locations: Locations 1 and 2 on the TM surface at (−23 mm, 0 mm, 0 mm) and (−23 mm, −11 mm, 0 mm), with Location 2 positioned opposite an electrode, and Locations 3 and 4 on the housing surface at (−27 mm, 0 mm, 0 mm) and (−27 mm, −11 mm, 0 mm), with Location 4 on an electrode. For all cases, we set Δu=0 mV and $r_{m}=\frac{0.1}{1.415}\;mm$.

Both Location 1 and Location 2 are at the surface of the TM. Location 2 is opposite one electrode. Both Location 3 and Location 4 are at the surface of the housing. Location 4 is at one electrode.

**Figure 14:**
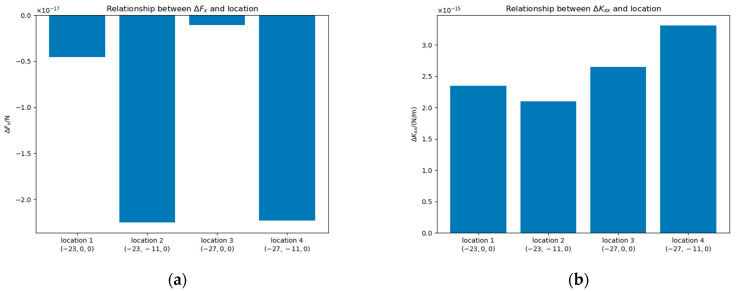
The values of $\Delta F_{x}$ (**a**) and $\Delta K_{xx}$ (**b**) at different locations of the contaminant bulge.

From Figure 14, it can be observed that patches opposite to an electrode or located at one electrode exhibit a bigger impact on $\Delta F_{x}$ compared to those situated in other regions. Conversely, patches on the housing surface demonstrate a significantly greater effect on $\Delta K_{xx}$ than those implemented on the TM surface.

### 4.3. Single Planar Patch Impact Analysis

In this section, we will investigate the influence of the variation of potential, size, and position of a single planar patch. The condition $d_{m}=0\;mm$ is adopted in Equation (8).

#### 4.3.1. Impact of Potential Δu

**Figure 15:**
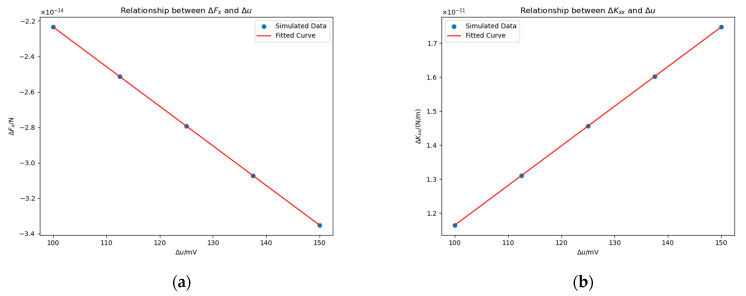
Impact of Δu on $\Delta F_{x}$ (**a**) and $\Delta K_{xx}$ (**b**) in the presence of a planar surface patch. Simulation parameters: the patch has a base radius of $r_{m}=0.5\;\mathrm{mm}$ and is centered at coordinates $\left(-23\;mm,\;0\;mm,\;0\;mm\right)$.

We vary Δu from 100 mV to 150 mV in steps of 12.5 mV, with $r_{m}=0.5\;mm$ and the patch centered at $\left(-23\;mm,\;0\;mm,\;0\;mm\right)$. Figure 15 shows $\Delta F_{x}$ and $\Delta K_{xx}$ under different Δu values. We fitted the data using a proportional function, as theoretically, when Δu=0 mV, both $\Delta F_{x}$ and $\Delta K_{xx}$ should equal zero. The fitted equations are:

(21) $$\Delta F_{x}\approx -2.23\times 10^{-16}\Delta u\;N,$$

(22) $$\Delta K_{xx}\approx 1.16\times 10^{-13}\Delta u\;N/m.$$

According to the result, we can conclude $\Delta F_{x}\propto \Delta u$ and $\Delta K_{xx}\propto \Delta u$.

**Figure 16:**
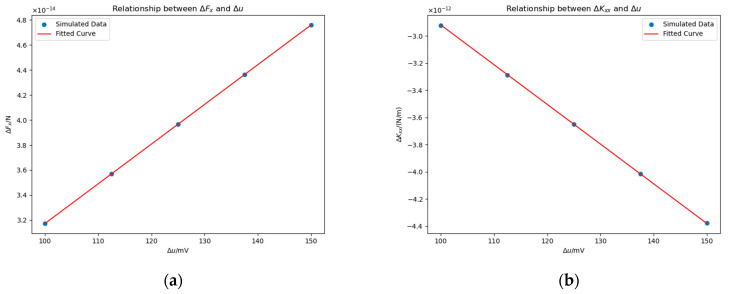
Effects of Δu on $\Delta F_{x}$ (**a**) and $\Delta K_{xx}$ (**b**) in the presence of a planar surface patch. Simulation parameters:$\;r_{m}=0.5\;mm;$ the patch is located at coordinates $\left(-23\;mm,-11\;mm,\;0\;mm\right)$.

To further validate this conclusion, we conducted additional experiments in which the patch was located at $\left(-23\;mm,-11\;mm,\;0\;mm\right)$ and $r_{m}=0.5\;mm$. Figure 16 shows the results, and the fitted proportional functions are given in Equations (23) and (24).

(23) $$\Delta F_{x}\approx 3.17\times 10^{-16}\Delta u\;N,$$

(24) $$\Delta K_{xx}\approx -2.92\times 10^{-14}\Delta u\;N/m.$$

The conclusion that $\Delta F_{x}\propto \Delta u$ and $\Delta K_{xx}\propto \Delta u$ has been further supported.

An additional observation is the reversal of monotonicity in $\Delta F_{x}$ and $\Delta K_{xx}$ between Figure 15 and Figure 16. This phenomenon will be explained in Section 4.3.3.

#### 4.3.2. Impact of Patch Radius $r_{m}$ and Position

To investigate the impact of patch radius $r_{m}$ and position on the electrostatic force and stiffness, we selected three distinct points, $\left(-23\;mm,-11\;mm,\;0\;mm\right),$ $\left(-23\;mm,\;3.5\;mm,\;0\;mm\right),$ and $\left(-23\;mm,\;0\;mm,\;0\;mm\right)$ as the center of the patch and conducted three sets of experiments. In each experiment, for each $r_{m}\in \left\{x|x=0.5k,k=1,2\ldots ,9,10\right\}\;mm$, we set Δu=100 mV and calculated $\Delta F_{x}$ and $\Delta K_{xx}$.

**Figure 17:**
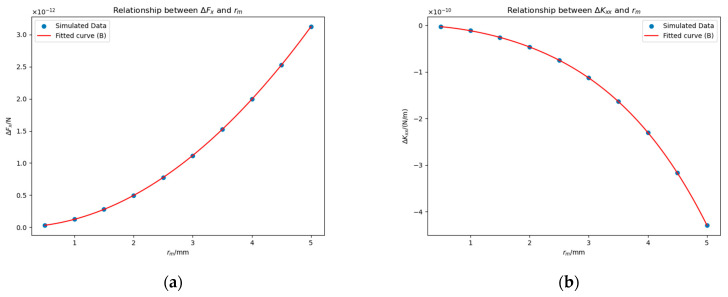
The values of $\Delta F_{x}$ (**a**) and $\Delta K_{xx}$ (**b**) under different $r_{m}$ when the patch is centered at coordinates $\left(-23\;mm,-11\;mm,\;0\;mm\right)$.

In the first set of experiments, the patch was centered at coordinates $\left(-23\;mm,-11\;mm,\;0\;mm\right),$ and the results are shown in Figure 17.

We tried to fit the data. Considering the accuracy of the fit and the simplicity of the expression, we obtained the following fitting results:

(25) $$\Delta F_{x}\approx \left(3.79\times 10^{-3}r_{m}^{3}+1.23r_{m}^{2}\right)\times 10^{-13}\;N.$$

(26) $$\Delta K_{xx}\approx \left(-4.74\times 10^{-1}r_{m}^{4}+1.48r_{m}^{3}-1.27\times 10^{1}r_{m}^{2}\right)\times 10^{-12}\;N/m.$$

**Figure 18:**
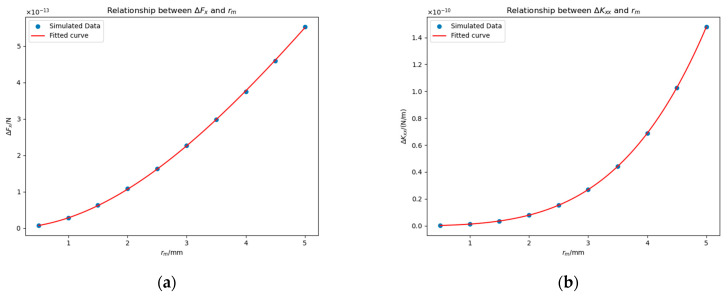
The values of $\Delta F_{x}$ (**a**) and $\Delta K_{xx}$ (**b**) under different $r_{m}$ when the patch is centered at coordinates $\left(-23\;mm,\;3.5\;mm,\;0\;mm\right)$.

In the second set of experiments, the patch was centered at coordinates $\left(-23\;mm,\;3.5\;mm,\;0\;mm\right),$ and the results are shown in Figure 18. We fitted the data and obtained the expressions:

(27) $$\Delta F_{x}\approx \left(-1.54r_{m}^{3}+2.97\times 10^{1}r_{m}^{2}\right)\times 10^{-15}\;N,$$

(28) $$\Delta K_{xx}\approx \left(1.50r_{m}^{4}+2.62r_{m}^{3}+8.49r_{m}^{2}\right)\times 10^{-13}\;N/m.$$

**Figure 19:**
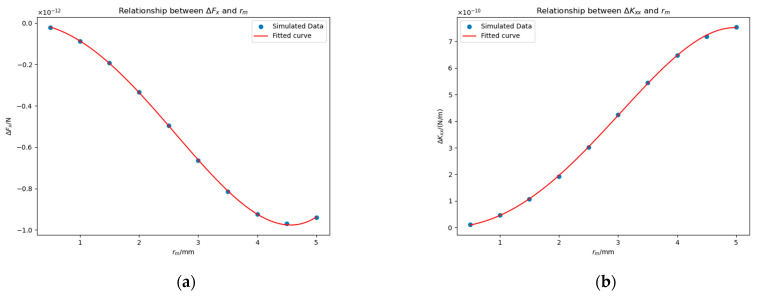
The values of $\Delta F_{x}$ (**a**) and $\Delta K_{xx}$ (**b**) under different $r_{m}$ when the patch is centered at coordinates $\left(-23\;mm,\;0\;mm,\;0\;mm\right)$.

In the third set of experiments, the patch was centered at coordinates $\left(-23\;mm,\;3.5\;mm,\;0\;mm\right),$ and the results are shown in Figure 19. We fitted the data and obtained the following expressions:

(29) $$\Delta F_{x}\approx \left(2.07\times 10^{-1}r_{m}^{4}+2.21\times 10^{-1}r_{m}^{3}-1.03\times 10^{1}r_{m}^{2}+1.23r_{m}\right)\times 10^{-14}\;N,$$

(30) $$\Delta K_{xx}\approx \left(-1.90r_{m}^{4}+6.42r_{m}^{3}+4.66\times 10^{1}r_{m}^{2}-5.85r_{m}\right)\times 10^{-12}\;N/m.$$

As can be readily observed from the results above, the changes in the electrostatic force and stiffness depend on both the position and size of the patch. When the patch is positioned directly opposite the electrode, we find that changes in electric forces and stiffness satisfy $\Delta F_{x}>0$, $\Delta K_{xx}<0$, given Δu>0, and the magnitudes of such changes increase with the patch radius $r_{m}$. When the patch is located opposite the non-electrode region of the housing, we find that $\Delta F_{x}<0$ and $\Delta K_{xx}>0$ for a given value of Δu>0, and the magnitudes of such changes increase with the patch radius $r_{m.}$ When the patch faces the edge of the electrode, the patch effects can be considered as the superposition of those from the above two cases. We will explain it in Section 4.3.3.

#### 4.3.3. Explanation of the Curve of $\Delta F_{x}$ and $\Delta K_{xx}$

**Figure 20:**
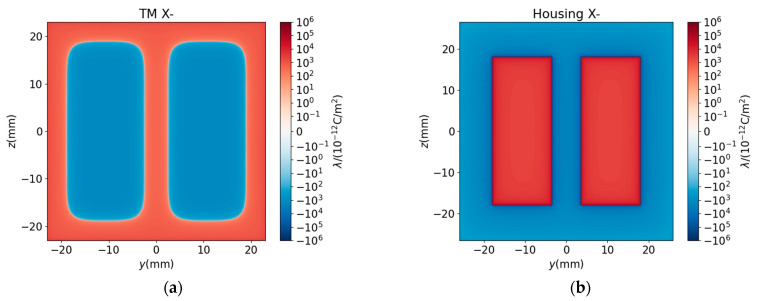
Distribution of charge density without patch. (**a**) TM surface at x=−23 mm. (**b**) Housing surface at x=−27 mm. Even though some part of the housing is grounded, this part still accumulates charges, just the potential is 0.

To explain the curve shape of $\Delta F_{x}$ and $\Delta K_{xx}$ mentioned above, we analyzed the charge redistribution induced by the patch and its interaction with the housing electrodes. Figure 20 illustrates the charge density (λ) distribution on the TM and housing surfaces.

**Figure 21:**
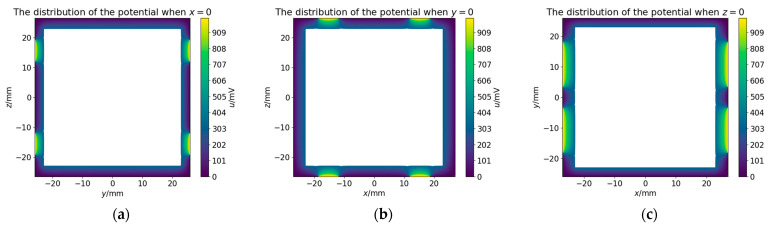
Equipotential plots of the electric potential distribution. (**a**) x=0; (**b**) y=0; (**c**) z=0.

To verify the charge distribution in Figure 20, we plotted the equipotential distribution on three planes (x=0, y=0, and z=0) derived from the charge distribution, as shown in Figure 21.

Figure 21 shows that: (i) the potential in the ground region of the housing is zero, (ii) the potential on the electrode matches the predefined potential value, and (iii) the TM is an equal potential body. These results thereby verify the correctness of the charge distribution.

Baseline Charge Distribution (Figure 20a,b): Under a 1000 mV bias applied to the sensing and actuation electrodes, the housing electrode regions exhibit strong positive charge accumulation, while the ground regions display negative charge accumulation (Figure 20b). Electrostatic coupling induces complementary charge polarization on the TM: negative charges dominate regions opposite housing electrodes, whereas positive charges accumulate elsewhere (Figure 20a).

**Figure 22:**
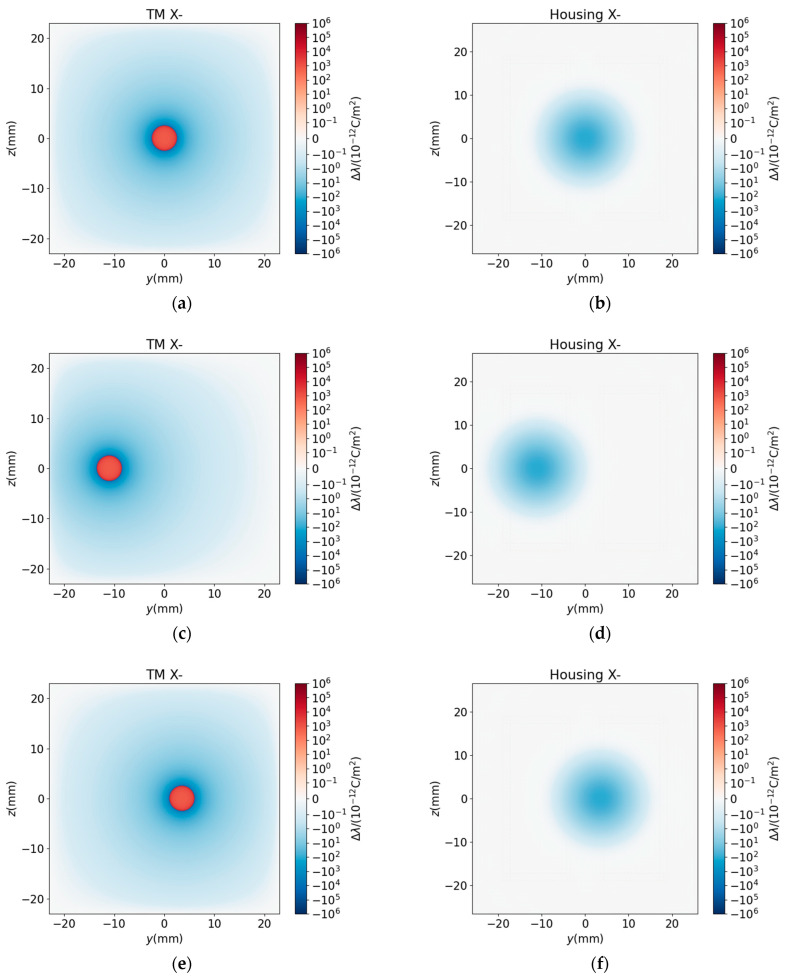
Distribution of changes in charge density under different circumstances. (**a**–**f**): $r_{m}=2.5\;mm$ and Δu=100 mV. (**a**,**c**,**e**): TM surface at x=−23 mm. (**b**,**d**,**f**): Housing surface at x=−27 mm. (**a**,**b**): A patch centered at $\left(-23\;mm,0\;mm,0\;mm\right)$. (**c**,**d**): A patch centered at (−23 mm, −11 mm, 0 mm). (**e**,**f**): A patch centered at $\left(-23\;mm,3.5\;mm,0\;mm\right)$.

Patch-Induced Charge Redistribution (Figure 22a–f): When a patch is present with Δu>0, additional positive charges accumulate within the patch region (Figure 22a,c,e), with negligible boundary effects due to the small boundary area. Additional negative charges accumulate on the surface of the housing opposite to the patch (Figure 22b,d,f).

From a physical point of view, $F_{x}$, $K_{xx}$ are determined by the interactions among the surface charges from the TM and housing. The dominant contributions are from the interactions between the baseline background charge distributions $\lambda_{TM}$ and $\lambda_{housing}$. The leading corrections arise primarily from interactions between the patch-induced charge and the original background, i.e., two pairs of interactions between ${\Delta \lambda }_{TM}$ and $\lambda_{housing}$ and between $\lambda_{TM}$ and $\Delta \lambda_{housing}$. As the magnitude of the patch-induced charge density is relatively small, the second-order corrections to the forces and stiffness from interactions between the patch-induced charge distribution can be ignored.

According to Equations (13) and (14), for the interaction between ${\Delta \lambda }_{TM}$ and $\lambda_{housing}$ ($\lambda_{TM}$ and $\Delta \lambda_{housing}$), if ${\Delta \lambda }_{TM}\lambda_{housing}>0$ ($\lambda_{TM}\Delta \lambda_{housing}>0$), then $\Delta F_{x}>0$ and $\Delta K_{xx}<0$; if ${\Delta \lambda }_{TM}\lambda_{housing}<0$ ($\lambda_{TM}\Delta \lambda_{housing}<0$), then $\Delta F_{x}<0$ and $\Delta K_{xx}>0$.

In Figure 15, the patch is opposite the non-electrode region and Δu>0; we observe that $\Delta \lambda_{TM}>0$ and $\Delta \lambda_{housing}<0$, leading to $\Delta F_{x}<0$ and $\Delta K_{xx}>0$. On the contrary, in Figure 16, the patch is opposite an electrode, Δu>0; we observe that $\Delta \lambda_{TM}<0$, $\Delta \lambda_{housing}$ > 0, resulting in $\Delta F_{x}>0$ and $\Delta K_{xx}<0$. The magnitude of these deviations scales linearly with Δu.

In Figure 17, no matter how $r_{m}$ changes, the patch is always only opposite an electrode, and Δu>0, so $\lambda_{TM}<0,\;\lambda_{housing}>0,\;\Delta \lambda_{TM}>0,\;\Delta \lambda_{housing}<0$, so $\Delta F_{x}>0$, $\Delta K_{xx}<0$. The curves become steeper and steeper because the area of the patch is a quadratic function of $r_{m}$.

In Figure 18, no matter what the value of $r_{m}$ is, half of the patch is always opposite an electrode, and the other half is not opposite. The shape of the curve is determined by the superposition of the effects of the two parts.

In Figure 19, Δu>0, which determines $\Delta \lambda_{TM}>0$ and $\Delta \lambda_{housing}<0$ when the patch is not big enough, it is only opposite the ground area on the housing, so $\lambda_{TM}>0,\;\lambda_{housing}<0$ and $\Delta F_{x}<0$, $\Delta K_{xx}>0$. The curve gets steeper and steeper. As $r_{m}$ expands further, a part of the patch will be opposite an electrode. In this part, $\lambda_{TM}<0,\;\lambda_{housing}>0$; the superposition of this part with the former causes the concavity, monotonicity, and steepness of the curves to be affected.

### 4.4. Analysis of the Metric Requirements in Space-Borne Gravitational Wave Detection Missions

The acceleration noise of inertial sensors can be classified into two types: direct noise and coupled noise. The direct noise of the patch field effect needs to be calculated based on a large amount of time series data related to the patch parameters, which has extremely high requirements for the efficiency of the simulation algorithm and computing power resources. This section does not conduct research on direct noise. Coupled noise is formed by the coupling of the position fluctuation of the TM and stiffness.

This section will respectively analyze the parameter limitations of bulges or large planner patches. In extreme cases, the stiffness of the patch field effect is approximately $3\times 10^{-9}\;s^{-2}$ [10]. In this section, we adopt a stricter value, with the stiffness of the patch not exceeding $1\times 10^{-10}\;s^{-2}$ (that is, the stiffness of the force is $1.93\times 10^{-10}\;N/m$ when the mass of the TM is 1.93 kg).

#### 4.4.1. Patch with Bulge

Figure 14 indicates that the change in stiffness at different positions is not significant. Therefore, we ignore the differences in the effects of bulges at different locations.

According to Equation (20), when Δu=0, $\Delta K_{xx}\approx 6.17\times 10^{-12}r_{m}^{3}\;N/m$. If we let $\Delta K_{xx}<1.93\times 10^{-10}\;N/m$ then we can obtain $r_{m}<3.15\;mm$. This is a very broad range. It can be considered that the impact of the bulge of a single patch can be ignored. If there are *N* bulges that do not affect each other, and the value of $r_{m}$ of each bulge is 0.1 mm, we can obtain $N<3.12\times 10^{4}$ according to $N\Delta K_{xx}<1.93\times 10^{-10}\;N/m$. Such a requirement is very lenient.

According to Equation (16), when $r_{m}=\frac{0.1}{1.415}\;mm$, $\Delta K_{xx}\approx \left(3.94\Delta u+2.35\right)\times 10^{-15}\;N/m$. If we assume there are *N* bulges that do not affect each other and let $\Delta u=150\;mV,N\Delta K_{xx}<1.93\times \frac{10^{-10}\;N}{m},$ then we can obtain N<325. Such restrictions can still be ignored.

#### 4.4.2. Single Planar Patch

In this section, we look for the values of Δu and $r_{m}$ that meet the stiffness limitation when the center of the patch is located at $\left(-23\;mm,-11\;mm,\;0\;mm\right)$ and $\left(-23\;mm,\;0\;mm,0\;mm\right)$, respectively.

**Figure 23:**
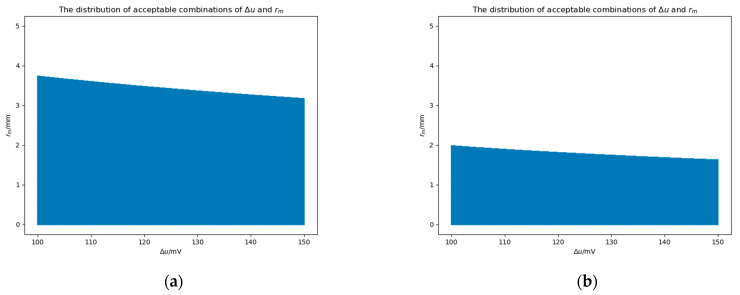
The distribution of acceptable combinations of Δu and $r_{m}$. (**a**) The center of the patch is $\left(-23\;mm,-11\;mm,\;0\;mm\right)$. (**b**) The center of the patch is $\left(-23\;mm,\;0\;mm,\;0\;mm\right)$.

Figure 23 shows the distribution of acceptable combinations of Δu and $r_{m}$. If the tuple $\left(\Delta u,r_{m}\right)$ is acceptable, the point will be marked as blue. With the increase in Δu, the acceptable values of $r_{m}$ in Figure 23a,b are gradually decreasing. When Δu increases to 150 mV, the acceptable values of $r_{m}$ in Figure 23a,b decrease to 3.16 mm and 1.62 mm, respectively. In a word, in order to ensure that the stiffness meets the requirements when $\Delta u\in \left[100\;mV,150\;mV\right]$, $r_{m}$ needs to be less than 1.62 mm.

## 5. Conclusions

In this paper, we provided a method that can be used to simulate the influences of a given patch field on the force and the stiffness on the TM and used this method to conduct some experiments. We can draw conclusions as follows:

1. We established a mathematical model based on a partition of boundary elements and the GMRES method to simulate the patch field. This model can solve the charge distribution according to the potential distribution and use the charge distribution to calculate the force and stiffness on the TM. The bulges and the nonuniform distribution of the potential led by patches can also be simulated with the model.
2. To overcome the difficulty of excessive computational complexity, based on existing algorithms, we made improvements according to needs, and designed an algorithm with $O\left(n\right)$ spatiotemporal complexity for calculating potential distribution, force, and stiffness.
3. With the method mentioned above, we researched what impacts single bulge forms from contaminant attachment have on force, $F_{x}$, and stiffness, $K_{xx}$. The control variable method is adopted, and Δu, $r_{m}$, and location are taken as variables, respectively. The results show that both $\Delta F_{x}$ and $\Delta K_{xx}$ are linear functions of Δu, approximately proportional to $r_{m}$ to the third power. The patch which is opposite one electrode or is at one electrode has a bigger impact on $\Delta F_{x}$ than the patch in the other area. The patch at the surface of the housing has a bigger influence on $\Delta K_{xx}$ than the patch at the surface of the TM.
4. In addition, we also studied a single patch without a bulge and found that the relation of $\Delta F_{x}$ and $\Delta K_{xx}$ to $r_{m}$ can be approximated to a quartic curve passing through the origin, which can be simplified in some cases, and both $\Delta F_{x}$ and $\Delta K_{xx}$ are approximately proportional to Δu.
5. With the help of Conclusion 3~4, we respectively studied the bulges and single planar patch for space-borne gravitational wave detection missions. If we limit the stiffness caused by the patch not to exceed $1.93\times 10^{-10}\;N/m$, we found that, under normal circumstances, the impact of a bulge can be ignored. When $\Delta u\in \left[100\;mV,150\;mV\right]$, the value of $r_{m}$ of a single patch should be less than 1.62 mm.

This study focuses on patch effects in inertial sensors and establishes a simulation framework that is scalable to the case where alternating current (AC) voltage is applied to actuation electrodes, as well as to the multi-patch case. Future research will focus on more complex scenarios and exploring mitigation strategies to reduce the impact of patch fields on gravitational wave detection. We will also try to study the general nonuniform distribution of electric potential caused by patch field effects based on the Monte Carlo method, rather than single patches, but this requires a large number of experiments to reach a statistically significant conclusion. Due to the large scale of the partition of our simulation experiment, we will further optimize the algorithm and improve the computing power resources to complete this part of the work. Our partner team is conducting patch effect experiments simultaneously, and there will be an actual measurement of the patch distribution in the future.

In addition to the patch field effects described in this article, other interference sources affecting the TM include temperature variations, residual gas interactions, cosmic ray exposure, self-gravitation force, magnetic fields, and so on. The influence of the patch field effects may exhibit dependencies on these factors. Moreover, temporal variations must be systematically taken into consideration. In future work, we will analyze the coupled interactions and incorporate time-varying analyses during satellite orbital operations.

## Figures and Tables

**Table 1:** Eight areas forming after the recursive process.

ID	Area	ID	Area
1	$\left[x_{0},x_{max}\right]\times \left[y_{0},y_{max}\right]\times \left[z_{0},z_{max}\right]$	5	$\left[x_{0},x_{max}\right]\times \left[y_{0},y_{max}\right]\times \left[z_{min},z_{0}\right]$
2	$\left[x_{min},x_{0}\right]\times \left[y_{0},y_{max}\right]\times \left[z_{0},z_{max}\right]$	6	$\left[x_{min},x_{0}\right]\times \left[y_{0},y_{max}\right]\times \left[z_{min},z_{0}\right]$
3	$\left[x_{min},x_{0}\right]\times \left[y_{min},y_{0}\right]\times \left[z_{0},z_{max}\right]$	7	$\left[x_{min},x_{0}\right]\times \left[y_{min},y_{0}\right]\times \left[z_{min},z_{0}\right]$
4	$\left[x_{0},x_{max}\right]\times \left[y_{min},y_{0}\right]\times \left[z_{0},z_{max}\right]$	8	$\left[x_{0},x_{max}\right]\times \left[y_{min},y_{0}\right]\times \left[z_{min},z_{0}\right]$

**Figure A1:**
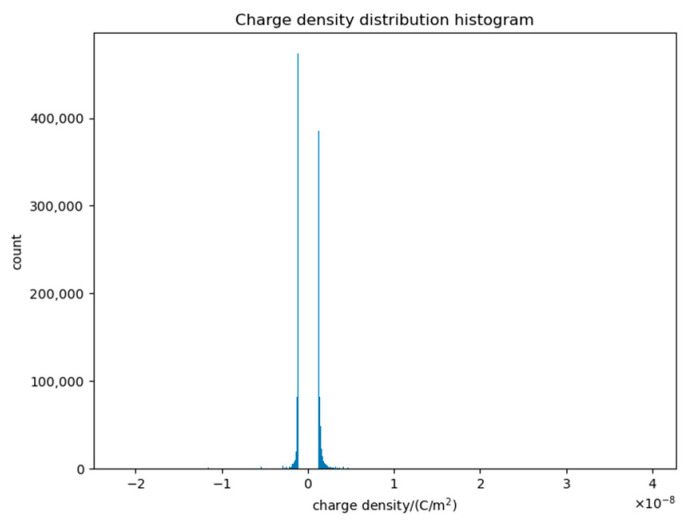
The charge density distribution histogram of each triangular element.

## Data Availability

The raw data supporting the conclusions of this article will be made available by the corresponding author on request.

## Appendix A. Verifying the Correctness of the Simulation Algorithm with a Parallel-Plate Capacitor

Before our experiments, we verified the correctness of our simulation algorithm by using a parallel-plate capacitor with plate spacing d and each plate being a square with side length l, ignoring the edge effect of capacitance. The value of the capacitance is

(A1) $$C=\frac{\epsilon_{0}l^{2}}{d}.$$

If one plate is grounded and the other plate is connected to VCC with a voltage of V, the amount of charge contained on a plate is

(A2) $$Q=\pm CV=\pm \frac{\epsilon_{0}l^{2}V}{d}.$$

The charge density on each plate is

(A3) $$\lambda =\frac{Q}{l^{2}}=\pm \frac{\epsilon_{0}V}{d}.$$

On the premise of ignoring the edge effect, the electric field between the plates is uniform, and the electric field intensity is

(A4) $$E=\pm \frac{V}{d}.$$

The electric field force on one plate is

(A5) $$F=\frac{E}{2}Q=\pm \frac{\epsilon_{0}l^{2}V^{2}}{2d^{2}}.$$

If we let l=50 mm, V=500 mV, and d=4 mm, then $\lambda \approx 1.11\times 10^{-9}\;C/m^{2}$ and $F\approx \pm 1.73\times 10^{-10}\;N$. Using our simulation algorithm, the electric field force on one plate is calculated to be $F\approx 1.78\times 10^{-10}\;N$, and the charge density distribution histogram of each triangular element is shown as Figure A1.

There is an error in the value of the electric field force, which is acceptable considering the existence of the edge effect. The value of charge density is mainly distributed around $-1.1\times 10^{-9}\;C/m^{2}$ and $1.1\times 10^{-9}\;C/m^{2}$, which is consistent with the theoretical value. The correctness of the simulation algorithm is verified.
